# Measurement of free glucocorticoids: quantifying corticosteroid-binding globulin binding affinity and its variation within and among mammalian species

**DOI:** 10.1093/conphys/cov020

**Published:** 2015-05-15

**Authors:** Brendan Delehanty, Sabrina Hossain, Chao Ching Jen, Graham J. Crawshaw, Rudy Boonstra

**Affiliations:** 1 Centre for the Neurobiology of Stress, Department of Biological Sciences, University of Toronto Scarborough, Toronto, Ontario, Canada M1C 1A4; 2 Toronto Zoo, 361A Old Finch Avenue, Toronto, Ontario, Canada M1B 5K7

**Keywords:** equilibrium dissociation constant, free hormone hypothesis, individual variation, laboratory methods

## Abstract

Plasma glucocorticoids (GCs) are commonly used as measures of stress in wildlife. A great deal of evidence indicates that only free GC (GC not bound by the specific binding protein, corticosteroid-binding globulin, CBG) leaves the circulation and exerts biological effects on GC-sensitive tissues. Free hormone concentrations are difficult to measure directly, so researchers estimate free GC using two measures: the binding affinity and the binding capacity in plasma. We provide an inexpensive saturation binding method for calculating the binding affinity (equilibrium dissociation constant, *K*_d_) of CBG that can be run without specialized laboratory equipment. Given that other plasma proteins, such as albumin, also bind GCs, the method compensates for this non-specific binding. Separation of bound GC from free GC was achieved with dextran-coated charcoal. The method provides repeatable estimates (12% coefficient of variation in the red squirrel, *Tamiasciurus hudsonicus*), and there is little evidence of inter-individual variation in *K*_d_ (range 2.0–7.3 nM for 16 Richardson's ground squirrels, *Urocitellus richardsonii*). The *K*_d_ values of 28 mammalian species we assessed were mostly clustered around a median of 4 nM, but five species had values between 13 and 61 nM. This pattern may be distinct from birds, for which published values are more tightly distributed (1.5–5.1 nM). The charcoal separation method provides a reliable and robust method for measuring the *K*_d_ in a wide range of species. It uses basic laboratory equipment to provide rapid results at very low cost. Given the importance of CBG in regulating the biological activity of GCs, this method is a useful tool for physiological ecologists.

## Teaser

Understanding how wildlife cope with stressors gives us insight into their potential impact. To quantify key hormones of stress - glucocorticoids – one needs to measure both how much there is in plasma (Total) and how much is not bound to a specific binding protein (Free). Here, we layout the first step in calculating FREE – measuring how strongly the specific protein binds the glucocorticoid.

## Introduction

Plasma glucocorticoid (GC) levels are commonly used to assess stress in wildlife. However, most vertebrates have corticosteroid-binding proteins that bind circulating GCs (Seal and Doe, 1965; Desantis *et al*., 2013), thereby limiting the ability of the GCs to leave the circulation and exert biological effects on tissues. Recent evidence from the biomedical literature supports the proposition that free hormone levels in plasma (i.e. the circulating hormone not bound to orticosteroid-binding globulin, CBG) are what the tissue really ‘sees’ (Qian *et al*., 2011). Given that CBG levels are dynamic (recently reviewed by Breuner *et al*., 2013) and that species vary considerably in binding capacity relative to their total plasma GC levels (Desantis *et al*., 2013), measurement of plasma free hormone levels is essential for understanding the biological significance of GC levels (Breuner *et al*., 2013). Nevertheless, the vast majority of studies on wild vertebrates have quantified only total GC concentrations, not free concentrations. Part of the reason for this may be the perceived difficulty in making the additional measurements needed to quantify free GC concentrations.

There are several methods available for measuring free hormone levels. Direct measurement of free hormone can be accomplished through ultrafiltration or dialysis (e.g. Lentjes *et al*., 1997). However, if the objective is to understand how an animal's hypothalamic–pituitary–adrenal axis is functioning, one needs to know not only the concentration of free GC, but also of total GC and CBG and how each of them changes over time (with season, reproductive condition and environmental stressors) or between experimental treatments. To realize this goal, we need to measure total GC levels, measure the GC-binding capacity of plasma CBG, and then estimate free hormone levels using a formula based on the laws of mass action (Barsano and Baumann, 1989).

**Figure 1:**
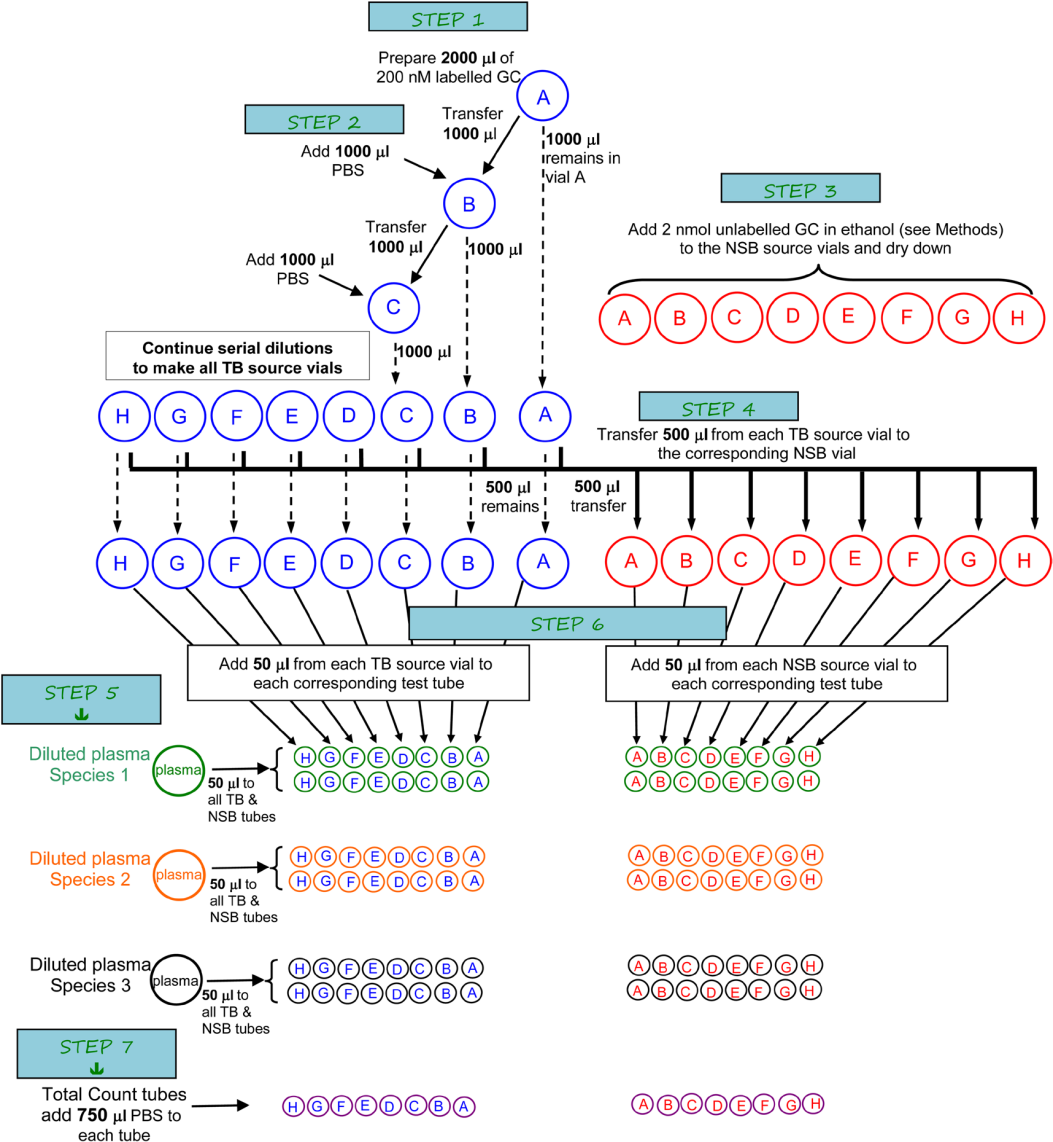
Illustration of the procedures for preparing source hormones and setting up the incubation of plasma and hormones for the saturation binding assay. The volumes indicated are sample volumes sufficient to run the assay with three species at once. Total binding (TB) vials and tubes are illustrated on the left; non-specific binding (NSB) vials and tubes are on the right. The process runs from top to bottom, and this figure is best understood in conjunction with the detailed assay steps spelled out in Box 1. Large circles are 20 ml scintillation tubes and small circles are 12 mm × 75 mm test tubes. Solid arrows indicate transferring or adding volumes; dashed lines indicate what is kept in the original vial. Abbreviations: GC, glucocorticoid (cortisol or corticosterone, depending on the study species); PBS, phosphate-buffered saline.

To use this formula, one must have measured the species' CBG equilibrium dissociation constant (*K*_d_), which represents the concentration of free hormone at which half of all CBG molecules are bound. A low *K*_d_ indicates a strong affinity of CBG for GC, whereas a high *K*_d_ indicates a weak affinity. For comparative physiologists, measurement of *K*_d_ is most readily achieved using saturation binding experiments, and three main methods have been used to accomplish this, namely microdialysis (e.g. Boonstra *et al*., 2001), filtration (e.g. Breuner and Orchinik, 2001) and charcoal separation (e.g. Hammond and Lahteenmaki, 1983). All three methods produce saturation curves from which *K*_d_ can be calculated. However, of these three methods, charcoal separation requires the least sophisticated equipment and is inexpensive to run (a standard run of three species or three plasma dilutions like that illustrated in Fig. 1 costs us less than $60 in consumables).

Our purpose in this paper is 2-fold. First, we want to provide a detailed account of how to run saturation binding assays using the charcoal separation method because the existing literature provides only bare outlines of the technique, and this has made it difficult for researchers to adopt the technique for quantifying *K*_d_ for their own study species. Second, we use the technique to investigate the degree of variability in *K*_d_ measurements among replicate assays, among individuals within a species and among 28 mammalian species. We will not address the issue here of whether there is any point in quantifying free GC levels at all (i.e. Schoech *et al*., 2013). The recognition that CBG plays a key role in understanding the impact and dynamics of GCs began in the 1950s shortly after the isolation and identification of GCs from the adrenal cortex, and by the mid-1960s major reviews were being published in both biomedicine (Sandberg *et al*., 1966) and wildlife (Seal and Doe, 1966). A recent assessment in biomedicine argues strongly that its measurement is essential (reviewed by Perogamvros *et al*., 2012), as do assessments in wildlife (Breuner *et al*., 2013; Wingfield, 2013), and a number of wildlife studies are interpretable only if free GC levels are known (e.g. Bradley *et al*., 1980; Boonstra *et al*., 1998; Romero *et al*., 2006).

## Materials and methods

Most vertebrates have either corticosterone (amphibians, reptiles, birds, some rodents and rabbits) or cortisol (most fish and mammals) as their dominant active GC (Sheriff *et al*., 2011). Corticosteroid-binding globulin binds both GCs, and the *K*_d_ is determined for the GC that is the dominant or sole GC in the plasma of the species of interest. Some species have both GCs in relative abundance for reasons that remain unexplored (e.g. Bradley, 1987; Delehanty, 2012), in which case the *K*_d_ must be determined for both GCs.

Our saturation binding protocol is a charcoal separation method based on that of Hammond and Lähteenmäki (1983), but modified to minimize the volume of plasma used and to avoid the need for a specialized nine-channel pipette. The article by Hammond and Lähteenmäki (1983) is a valuable resource, especially in its consideration of competition between sex hormone-binding globulin and CBG for the ligand, but it can be difficult to use as practical guide to running saturation binding assays for those not familiar with the principles and techniques. To provide a simple entry point for researchers not familiar with these techniques, Box 1 provides step-by-step details of our methods, Fig. 1 provides a visual guide to preparing the assay, and Supplementary Appendix 1 provides a spreadsheet that helps with calculations used in the assay, can be used for data entry and provides examples of output.

**Box 1:** Most published equilibrium saturation binding methods assume a high degree of familiarity with the technique or require piecing together the methods from a series of papers. In this Box, we provide detailed instructions, which, in conjunction with the spreadsheet provided in Supplementary Appendix 1, will allow researchers with little previous experience in these assays to calculate the dissociation constant (*K*_d_) for their species of interest. Figure 1 provides a visual aid for understanding the assay procedures.

**Advance preparations**

Before commencing the assay, we prepare radioactively labelled and unlabelled glucocorticoid (GC) stock solutions. The labelled [1,2,6,7-^3^H]GC (Perkin Elmer, Waltham, MA, USA) is received in ethanol solvent, which we first dilute 20-fold with redistilled ethanol; this is our source solution. It is stored in a freezer at −20°C. Unlabelled GC is prepared by dissolving ∼10.0 mg of GC (Steraloids Inc., Newport, RI, USA) in an equivalent volume of redistilled ethanol to produce a 1.0 mg ml^−1^ solution. This solution is repeatedly vortexed and allowed to dissolve overnight at 4°C. We use this to prepare a 0.1 mg ml^−1^ solution, also with redistilled ethanol. We store these ethanol solutions in a freezer at −20°C for up to several months. Note that redistillation of the ethanol ensures absolute purity, because peroxides can develop over time when the ethanol is exposed to air in the bottle at room temperature.

We prepare dextran-coated charcoal (DCC) concentrate by adding 100 ml of ultrapure water to 6.25 g of activated charcoal (Sigma, Activated C5260; Norit A) in a graduated cylinder dedicated to charcoal preparation (we find it impossible to remove all traces of charcoal from the glassware), covering with Parafilm (Pechiney Plastics Packaging, Menasha, WI, USA) and shaking vigorously. After allowing the charcoal to settle for ≥2 h, the supernatant is decanted, new water is added, and the process is repeated several times. This serves to remove fine charcoal particles that would not settle quickly when centrifuged. After the final decanting, we add 0.625 g of Dextran T-70 (Pharmacia Fine Chemicals) and add ultrapure water to a final volume of 100 ml. This is shaken well to ensure that the dextran is dissolved and stored at 4°C for up to several weeks. Solutions are prepared with phosphate-buffered saline [PBS; 8.66 g Na_2_HPO_4_ (anhydrous), 6.10 g NaH_2_PO_4_.2H_2_O, 1 g gelatin and 0.1 g thimerosal (as a bacteriostatic preservative; Sigma T8784, Oakville, Ontario, Canada)]. We store PBS at 4°C for up to 1 week.

For the plasma samples, we generally begin with plasma pools (plasma from several, preferably more than five, animals of both sexes mixed together) of ∼1 ml, from which we strip endogenous steroids by adding ∼70 mg activated charcoal in a 1.5 ml microtube. We vortex the tube, incubate it at 37°C for 4 h, then we repeatedly centrifuge and transfer the supernatant to a new microtube to ensure that no residual charcoal is present in the stripped plasma. We usually recover ∼800 μl of stripped plasma for every 1 ml of plasma pool. Stripped plasma is stored in a freezer at −20°C if it is going to be used within a month, or at −80°C for longer-term storage. None of the above solutions stored at −20°C should be stored in a frost-free freezer because these undergo brief thaw cycles daily, and plasma stored in such conditions degrades rapidly.

**Assay preparations**

The saturation binding assay uses the following three sets of tubes:

- Total binding (TB) tubes; these receive plasma (stripped of endogenous GC) and a dilution series of radioactively labelled GC. When measuring the amount of bound labelled GC in these tubes [measured in disintegrations per minute (dpm) in a scintillation counter], one is measuring binding by both CBG and non-specific binding proteins.
- Non-specific binding (NSB) tubes; these receive plasma, the same dilution series of labelled GC as in the TB tubes, plus a constant concentration of unlabelled GC greatly in excess of CBG binding capacity. Given that CBG has limited binding capacity and because there is far more unlabelled GC than labelled GC in these tubes, only the high-capacity non-specific binding proteins (primarily albumin) end up binding labelled GC. Thus, the dpm measured in these tubes are measures only of non-specific binding.
- Total count tubes; these receive only radioactively labelled GC and buffer. The purpose is to obtain a measure in dpm of the amount of GC that has been introduced into the previous two sets of tubes in order to calibrate the dpm values with a known amount of labelled GC.

Preparation of the labelled GC dilution series needs to begin 2 days prior to running the assay. For most of our work, we assume that *K*_d_ values will be of the order of 1–20 nM and that a maximal concentration of 100 nM of GC will be likely to produce an asymptote. Given that the plasma and GC solutions are added in equal volumes in the assay, a final concentration of 100 nM requires preparation of a 200 nM starting concentration of labelled CG (Step 1 in Fig. 1; concentration A).

Given that the highest concentration source solution is used to make all the serial dilutions for the TB, NSB and total count tubes, we prepare four times the final volume needed. For a run of three species, we needed six tubes (three species × two replicates) for each TB and NSB concentration (A–H), plus one set of TB and NSB total count tubes (Fig. 1). We use 50 μl of source solution per tube, so we need at least 350 μl source solution for each concentration. To ensure ease of pipetting and provide a margin of safety, we prepare 500 μl of each TB and NSB source concentration. Thus, we need to prepare 2000 μl of the initial 200 nM labelled GC (Step 1 in Fig. 1). This quantity can be scaled up or down based on the number of runs planned, but all further references to volumes of source solutions are based on this standard run. Preparation of the 200 nM labelled GC requires knowledge of the specific activity of the labelled GC. The spreadsheet in Supplementary Appendix S1 includes a worksheet to assist with this calculation. Thus, for example, our labelled cortisol is generally 79 Ci mmol^−1^, which requires 632 μl of our (diluted) source solution. After adding the labelled GC to a 20 ml glass scintillation vial, we dry it under filtered air [i.e. ordinary laboratory air supply passed through a Millipore filter (Millex Vent Filter Unit FG 0.2 μm; Millipore Corporation, Milford, MA, USA)]. We then add 2000 μl PBS and allow it to reconstitute overnight at 4°C. In the past, we dried steroid under nitrogen gas to avoid oxidation of labelled GC, but we have not detected any detriment to using filtered air. However, researchers wishing to avoid the risk of oxidation could consider adding labelled GC directly to PBS, as long as the total volume of solvent (ethanol in our case) is minute relative to the volume of PBS. If using this method, it would be preferable to skip the 20-fold dilution of the labelled GC we outlined in ‘*Advance preparations*’.

The next morning, we prepare the TB source dilutions (Step 2 in Fig. 1). We thoroughly vortex the 200 nM solution (100 nM final dilution; concentration A in Fig. 1) and then make seven sequential 50% dilutions (concentrations B–H), down to 0.78 nM (final dilution; all further references to concentrations refer to the final concentration). Each serial dilution is made by transferring 1000 μl of the higher concentration into a new 20 ml glass scintillation vial and adding 1000 μl PBS (Step 2 in Fig. 1).

To prepare 500 μl of the NSB source solutions, we add 7.2 μl of 0.1 mg ml^−1^ unlabelled cortisol or 6.9 μl of 0.1 mg ml^−1^ unlabelled corticosterone in ethanol (Step 3 in Fig. 1). We dry down the ethanol in the NSB source solution vials, and then transfer 500 μl of the TB source solution vials to the corresponding NSB vials (Step 4 in Fig. 1). All vials are vortexed, and the NSB vials are allowed to reconstitute fully overnight at 4°C.

**Performing the assay**

On the morning of the assay, we first prepare the plasma dilutions using PBS (Step 5 in Fig. 1). We typically start with plasma dilutions of 1/50, but good saturation curves have required dilutions that range from 1/10 to 1/200, depending on species. Selecting the best dilution is a trial-and-error process; the goal is to have a dilution in which there is as little ligand depletion as possible. Ligand depletion occurs when a significant proportion of the hormone is bound by CBG at equilibrium. A good dilution should result in CBG saturation by only a small fraction of the added hormone, leaving most of the added hormone free.

Next, we prepare 12 mm  × 75 mm polypropylene tubes. For each species, we have 16 TB tubes and 16 NSB tubes (labelled concentrations A–H in duplicate), into which we add 50 μl of diluted plasma. The total count tubes do not receive any plasma. Next, using an electronic repeater pipette with 16 separate tips (one for each TB and NSB concentration) we add 50 μl of TB source solutions A–H to each species' TB tubes and one set of the total count tubes, and we add 50 μl of the NSB source solutions A–H to each species' NSB tubes and the other set of total count tubes (Step 6 in Fig. 1). Finally, we add 750 μl of PBS to the total count tubes (Step 7 in Fig. 1).

We cover and incubate the tubes at 37°C for ≥4 h on a rocking shaker. During this time, we chill a refrigerated centrifuge (Beckman Coulter, Allegra 6R) to 0°C. This centrifuge has four bucket positions, and each plastic bucket insert has 37 positions to hold the test tubes (centrifuge capacity is a key determinant of maximal run size). We prepare a saline or alcohol ice slurry (ice chips mixed with water and table salt or ethanol to lower the temperature of the slurry to 0°C) in a bin large enough to receive the tube racks. We prepare enough DCC in a beaker (72 ml PBS and 8 ml of DCC concentrate) for each TB and NSB tube to receive 700 μl diluted DCC. The DCC is kept in an ice slurry and stirred constantly using a magnetic stirrer. We use ice slurries to minimize the loss of CBG-bound hormone to the DCC. As free hormone is adsorbed by charcoal, more GC will come off the CBG, and this process is faster at higher temperatures; by keeping the temperature low, we minimize this loss.

At the end of the incubation period, the tube racks are placed in the ice slurry and allowed to chill for 5 min. We then rapidly add 700 μl of DCC to each of the TB and NSB tubes (but not to the total count tubes) using a manual repeater pipette (electronic repeater pipettes are too slow). The DCC is added to all tubes within 1 min, and a 10 min DCC exposure time starts once the last tube receives DCC. During that 10 min, the tubes are covered with plastic cling film and trays are vortexed briefly before being quickly transferred into the chilled centrifuge buckets and then to the centrifuge to continue their DCC exposure at 0°C. At the end of the 10 min, the tubes are spun at 2000***g*** for 12 min.

While centrifuging the samples, we add 2.5 ml of Biosafe II liquid scintillation cocktail fluid (Biosafe II; Research Products International, USA) to 6 ml polypropylene scintillation vials (Pony Vials; Perkin Elmer, Woodbridge, Ontario, Canada). Once samples are centrifuged, we transfer 500 μl of the supernatant to the scintillation vials, vortex, and then place the vials in a scintillation counter (Tri-Carb 2900TR; Packard, Boston, MA, USA). We allow the vials to sit in the dark in the counter for 4 h before counting, because we find that counts are more uniform if allowed some equilibration time.

**Data analysis**

**Figure 2:**
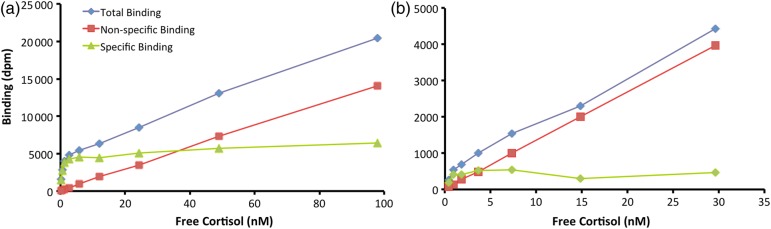
Examples of successful (a) and unsuccessful saturation binding curves for elk (b). The specific binding curves are calculated by subtracting the non-specific binding values from the total binding values. The non-specific binding line should be linear, while the total binding curve should be curvilinear, with the upper portions of the curve eventually matching the slope of the non-specific binding curve. That will produce the asymptote in the specific binding curve (a). The assay can fail for a variety of reasons, including bacterial contamination of buffer, imprecise pipetting or being too slow adding the charcoal. The *x*-axis is the amount of free GC in the test tube at equilibrium, which is not necessarily equal to the amount of labelled GC initially added. See Box 1 and Supplementary Appendix 1 for the method to calculate the *x*-values. The data from the specific binding curve are then fitted using non-linear regression to yield the estimate of *K*_d_.

Saturation binding data are analysed by plotting the amount of specific binding [total binding (dpm) minus non-specific binding (dpm) = specific binding (dpm)] as a function of the concentration of free hormone at equilibrium (e.g. Fig. 2a). Ideally, the concentration of free hormone will be almost equal to the total concentration of hormone added. The goal should be to have sufficiently diluted the plasma that at the lowest GC concentrations, >90% of labelled GC remains free. If this is the case, binding is typically plotted as a function of GC added. When more than 10% of GC ends up bound (an arbitrary threshold that appears in the literature without, to our knowledge, any empirical basis), ligand depletion becomes a concern for the data analysis. Given that we were working with a large number of species, we often obtained results that produced good saturation curves that nonetheless did not meet this threshold. We therefore used the total count tubes to calculate the real amount of free hormone in each vial {i.e. equilibrium free hormone concentration = [1 − (total binding counts/total counts)] × starting concentration; see Supplementary Appendix 1}. We then used non-linear regression (SAS Proc NLIN) to solve the equation: specific binding = *B*_max_ × [free hormone concentration/(free hormone concentration + *K*_d_)]. The input data are the specific binding (in dpm) and free hormone concentration (nanomolar), and the regression estimates *K*_d_ and maximal binding capacity (*B*_max_, measured in dpm).

### Basic principles of the assay

The goal of the saturation binding assay is to plot the amount of specific binding (i.e. binding by CBG, which has high affinity for GC but low capacity of only one GC molecule per CBG molecule; Hammond *et al*., 1991) over a wide range of GC concentrations, ranging from less than to manyfold higher than the expected *K*_d_ (a good rule of thumb is a range of concentrations from 1/10 to 10 times the expected *K*_d_). The resulting curve should reach an asymptote (Fig. 2), indicating that the CBG has been saturated. One can then derive the *K*_d_ from these data by fitting a curve using non-linear regression.

The specific binding is difficult to measure directly because proteins other than CBG also bind GCs. The most abundant plasma protein is albumin (Peters, 1995), which binds GCs with low affinity but very high capacity. This non-specific binding must be factored out in order to calculate the specific binding by CBG. This is accomplished by measuring the binding twice: first, total binding (both specific and non-specific) is measured by adding enough radioactively labelled GC (either [1,2,6,7-^3^H]corticosterone or [1,2,6,7-^3^H]cortisol, depending on the sole or predominant plasma glucocorticoid) to just saturate the CBG in the plasma sample; and second, non-specific binding is measured by adding so much unlabelled GC in addition to the radioactively labelled GC that only the virtually unsaturable non-specific binding proteins are likely to bind any of the labelled GC. By subtracting the amount of non-specific binding (NSB) from the total binding (TB) at each GC concentration in the dilution series, one can calculate the amount of specific (CBG) binding by CBG (Fig. 2).

An essential component of all saturation binding assays is the separation of bound from free hormone. In both TB and NSB tubes, we want to measure how much bound hormone there is in each tube at equilibrium, which means that we need to remove all of the free hormone, but only the free hormone, so that the remaining radioactivity is a measure of binding. Our assay uses dextran-coated charcoal (DCC) to accomplish the separation of bound from free hormone. The DCC adsorbs free hormone, but not bound hormone, so that when DCC is added to the plasma and the plasma is centrifuged, the supernatant contains CBG-bound hormone and the charcoal pellet contains the adsorbed free hormone. The radioactive content of the supernatant is then measured, and the saturation curve can be prepared.

A note of caution regarding the charcoal technique is warranted. Researchers must be aware that the removal of plasma free GC by charcoal disturbs the equilibrium and therefore CBG-bound GC will tend to be freed from CBG in order to establish a new equilibrium. The newly freed GC is then adsorbed by the charcoal, further shifting the equilibrium. This process is faster at higher temperatures and becomes more prominent as the length of the charcoal exposure increases. If the separation is not carried out quickly enough, a significant portion of GC that was bound by CBG in the original equilibrium conditions will be lost to the charcoal, thereby underestimating the real binding capacity of plasma CBG. To reduce this effect, charcoal-based separation methods must be carried out at low temperatures (∼0°C) and as quickly as possible. Our techniques for managing this are more fully explained in Box 1.

### Data analysis

Saturation binding data are analysed by comparing the amount of specific binding [i.e. specific binding (measured in disintegrations per minute, dpm) = total binding dpm minus non-specific binding dpm] as a function of the concentration of unbound hormone at equilibrium. We used non-linear regression (SAS Proc NLIN) to estimate the *K*_d_ and maximal binding capacity (*B*_max_, measured in dpm) in the following equation: specific binding = *B*_max_ × [free hormone concentration/(free hormone concentration + *K*_d_)]. To be clear, although this assay involves solving for *B*_max_, researchers will typically want to measure maximal binding capacity (a measure of the amount of CBG in plasma) in individual samples rather than from pooled samples. Scatchard plots of the saturation data are commonly used to calculate the *K*_d_ in the older literature, but relatively small errors in the saturation curve data can be magnified through the Scatchard approach (Motulsky and Ransnas, 1987). The ease with which non-linear regression can be performed on modern computers makes Scatchard plots obsolete.

### Variability among replicate runs

**Figure 3:**
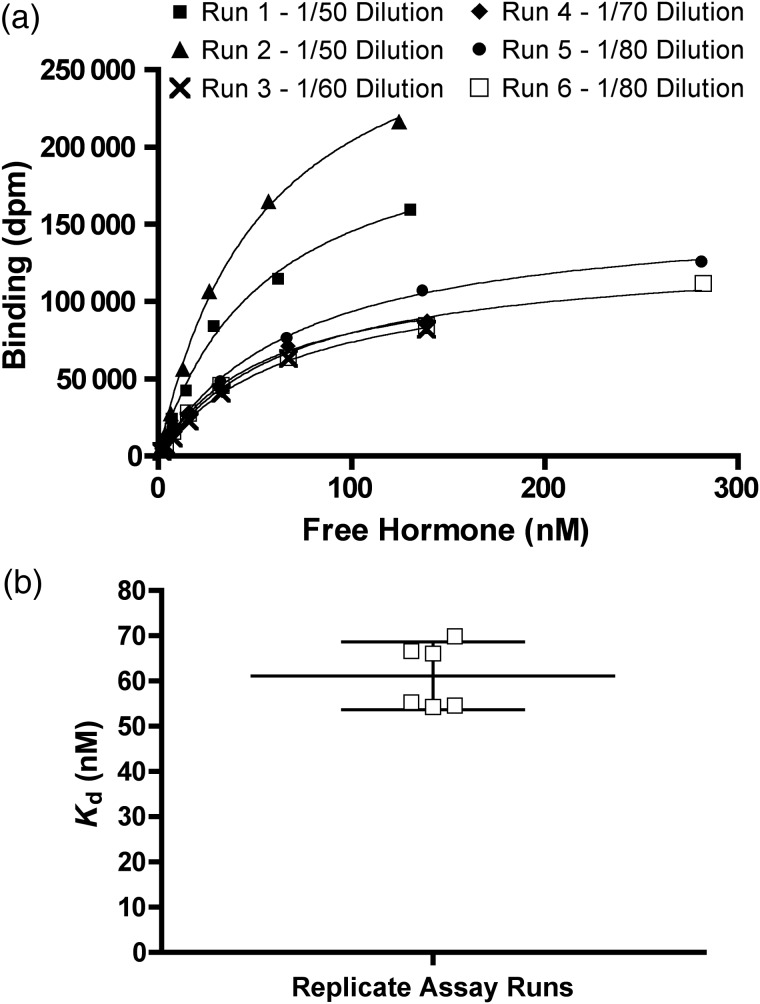
Saturation binding curves and *K*_d_ values for red squirrel plasma. The results of six runs using uniform pooled plasma are shown in (a) and the distribution of *K*_d_ values calculated from these runs, with mean (horizontal line) and 95% confidence intervals are shown in (b).

To test whether the same plasma sample produced the same *K*_d_ value, we ran repeated saturation binding assays on identical aliquots of pooled red squirrel (*Tamiasciurus hudsonicus*) plasma (from mixed sex and season samples, all collected from the same locality in the Yukon). After the initial pooling of plasma, each aliquot was stored separately so that the number of freeze–thaw cycles was the same for each run. After discarding some runs based on poor fit that we attributed to experimental error, we were left with six replicate runs (Fig. 3).

### Variability among individuals

To test whether different individuals within a species produced the same *K*_d_ value, we used Richardson's ground squirrel (*Urocitellus richarsonii*) samples from males either before or immediately after the spring breeding season. The samples were collected over 3 years in two geographical locations. After discarding some runs based on poor fit that we attributed to experimental error, we were left with 16 runs. Four samples were collected in 2007 from the University of Alberta Kinsella Ranch, Alberta (53° 0′ N, 111° 31′ W), four samples were collected near Moosomin, Saskatchewan (50° 6′ N, 101° 41′ W), and eight samples were collected from the Kinsella Ranch in 2009.

### Variability among mammalian species

To assess *K*_d_ values over a range of five mammalian orders and 28 species, we used plasma from our own fieldwork on a variety of small mammals (snowshoe hares, sciurids and microtine rodents) and serum from the Toronto Zoo. Where possible, we used pooled samples from a number of individuals, but most of the zoo samples came from single individuals. Details on the origins of the plasma/serum are set out in Table 1.

**Table 1:** Equilibrium dissociation constants for our study species, in order of increasing dissociation constant

Common name	Species	Source	GC	Dilution	*K*_d_ (nM)	SEM	Comments
Barbary sheep	*Ammotragus lervia*	Zoo	F	1/20	0.4	0.07	
Alpaca	*Vicugna pacos*	Zoo	F	1/50	0.6	0.15	
Snowshoe hare	*Lepus americanus*	Yukon	F	1/25	0.6	0.18	Plasma pool (males)
Western grey kangaroo	*Macropus fulginosus*	Zoo	F	1/50	0.7	0.03	
Elk	*Cervus canadensis*	Zoo	F	1/50	0.7	0.19	
Gaur	*Bos gaurus*	Zoo	F	1/50	0.7	0.16	
Siberian tiger	*Panthera tigris altaica*	Zoo	F	1/50	0.7	0.19	
Arctic wolf	*Canis lupus*	Zoo	F	1/50	0.8	0.11	Pooled from four animals
Bactrian camel	*Camelus bactrianus*	Zoo	F	1/50	1.0	0.16	
Soay sheep	*Ovis aries*	Scotland	F	1/50	1.7	0.55	Plasma pool
Collared lemming	*Dicrostonyx groenlandicus*	NWT	B	1/50	2.4	0.36	
Red river hog	*Potamochoerus porcus*	Zoo	F	1/50	3.3	0.11	
Arctic ground squirrel	*Urocitellus parryii*	Yukon	F	1/50	4.0	0.4	Mean of four runs on males
Grizzly bear	*Ursus arctos horribilis*	Zoo	F	1/50	4.0	0.23	
Spotted hyena	*Crocuta crocuta*	Zoo	F	1/50	4.0	0.4	Mean of two runs
Richardson's ground squirrel	*Urocittelus richardsonii*	Alberta	F	1/50	4.3	0.36	
Deer mouse	*Peromyscus maniculatus*	Ontario	B	1/200	4.9	0.61	Plasma pool
Columbian ground squirrel	*Urocitellus columbianus*	Alberta	F	1/50	5.1	1.0	Mean of five runs of male and female pools
Meadow vole	*Microtus pennsylvanicus*	Ontario	B	1/50	5.4	1.2	Mean of two runs, pooled plasma
Polar bear	*Ursus maritimus*	Zoo	F	1/50	6.2	1.20	
Barbary ape	*Macaca sylvanus*	Zoo	F	1/50	6.6	0.96	
Lynx	*Lynx canadensis*	Zoo	F	1/50	6.8	0.63	
Cheetah	*Acinonyx jubatus*	Zoo	F	1/50	8.0	2.40	
Thirteen-lined ground squirrel	*Ictidomys tridecemlineatus*	Manitoba	F	1/10	13.3	0.85	Plasma pool (males)
Olive baboon	*Papio anubis*	Zoo	F	1/50	15.3	0.76	
Franklin's ground squirrel	*Poliocitellus franklinii*	Manitoba	F	1/20	22.2	3.30	Plasma pool (males)
Eastern chipmunk	*Tamias striatus*	Ontario	F	1/50	42.9	2.20	Plasma pool
Red squirrel	*Tamiasciurus hudsonicus*	Yukon	F	1/80–1/10	61.1	2.90	Mean of six runs, pooled plasma

Serum was used in the assays for the zoo species; plasma was used for the assays for other species. Species are ranked according to dissociation constant (*K*_d_; from strongest binding to weakest). The glucocorticoid (GC) hormones used for the assays were cortisol (F) or corticosterone (B). Abbreviation: NWT, Northwest Territories, Canada.

## Results

### Variation among replicate runs

In our replicate red squirrel runs, we used various plasma dilutions (ranging from 1/50 to 1/80). We also increased the maximal cortisol concentrations used from 150 to 300 nM in an attempt to identify an asymptote better (Fig. 3a). The mean *K*_d_ was 61.1 nM [95% confidence interval (CI) 53.6–68.6 nM; Fig. 3b]. The inter-assay coefficient of variation was 12%.

### Variation among individuals

**Figure 4:**
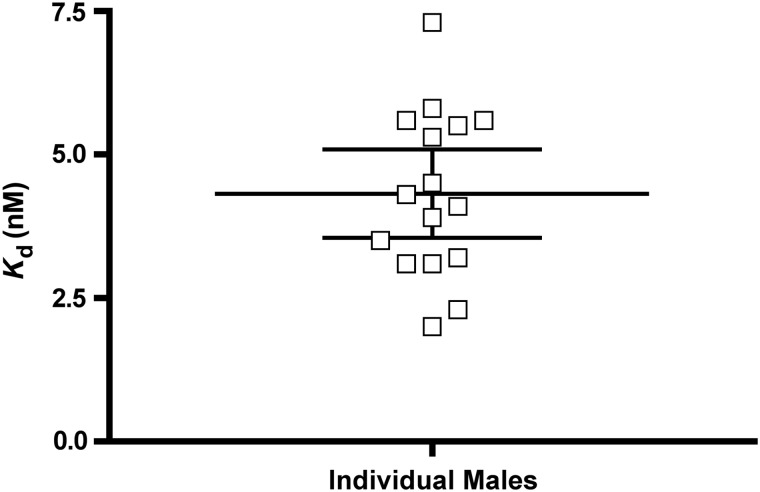
Plot of individual *K*_d_ values for male Richardson's ground squirrels with mean (horizontal bar) and 95% confidence intervals.

The mean *K*_d_ for all individual Richardson's ground squirrel males was 4.3 nM (95% CI 3.5–5.1 nM; Fig. 4). We found no effect of year or location on *K*_d_ (SAS Proc GLM, *F*_2,13_ = 0.64, *P* = 0.54). The *K*_d_ estimates from individuals ranged from 2.0 to 7.3 nM, with a coefficient of variation of 33%.

**Figure 5:**
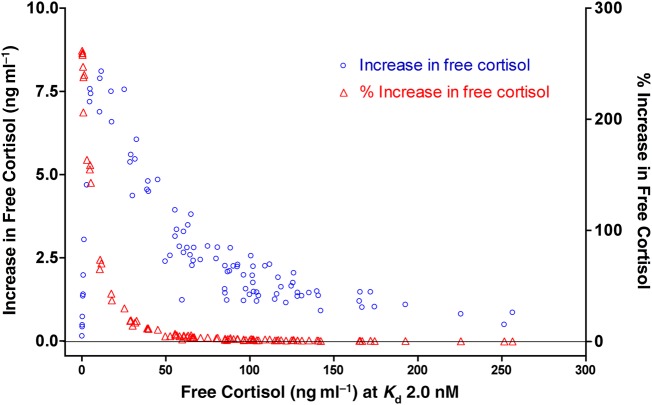
Given that our estimates of *K*_d_ for individual Richardson's ground squirrels ranged from 2.0 to 7.3 nM, we wanted to determine whether this difference was likely to have a significant effect on free hormone levels. This depends on the real GC concentrations and CBG capacity of individuals, so we used those data from 96 Richardson's ground squirrels studied in our previous studies (Delehanty and Boonstra, 2009, 2012), which had been measured in baseline or acute stress conditions. The figure shows the absolute (circles; left *y*-axis) and percentage increase (triangles; right *y*-axis) in free GC when the *K*_d_ is increased from 2.0 to 7.3 nM.

In looking at individual variation in *K*_d_ values, we are interested in determining whether it is important to know individual *K*_d_ values when calculating free cortisol levels or whether a mean value is sufficient. To determine the biological relevance of *K*_d_ variation (i.e. to determine how free cortisol levels are affected by variation in *K*_d_), one must know the ranges of total cortisol concentrations and cortisol-binding capacity that are found in nature. We used the total cortisol and binding capacity values from our previous studies (Delehanty and Boonstra, 2009, 2012) to calculate the difference in the amount of free hormone when *K*_d_ = 2.0 nM compared with *K*_d_ = 7.3 nM (Fig. 5). The maximal increase in free cortisol as a result of lowering the binding affinity was 8 ng ml^−1^, but for most animals the effect was <3 ng ml^−1^. The greatest changes in absolute and percentage terms occurred in animals that had low free cortisol levels (<50 ng ml^−1^), although the highest percentage increases were found among animals that had very low (<1 ng ml^−1^) free cortisol levels and so their absolute increases in free cortisol were also very small. In animals with >50 ng ml^−1^ free cortisol at *K*_d_ = 2.0 nM, the effect of increasing *K*_d_ was small in both absolute (generally <3 ng ml^−1^) and percentage terms (<10%).

### Variation among mammalian species

Using our charcoal separation technique, we were able to measure *K*_d_ for most of the species from which we had serum or plasma (Table 1). The *K*_d_ values ranged from 0.4 to 61 nM, with the majority of species having *K*_d_ values clustered at <8 nM.

We were unable to obtain good saturation binding curves for some of the species we attempted, and we were unable to obtain a saturation curve for binding of corticosterone by the plasma of Franklin's ground squirrels and thirteen-lined ground squirrels, which have substantial amounts of both cortisol and corticosterone circulating (data not shown). The assay needs to be optimized for each species (see Materials and methods), and because we were focused on the breadth of species being sampled, if we could not obtain a reasonable saturation curve within several attempts, we put our limited time and budget into another species.

## Discussion

### Variation among replicate runs within 'red squirrels

After eliminating runs in which experimental error was subjectively determined to have resulted in a poor curve (see Fig. 2 for examples of good and poor curves), the replicate runs all provided reasonably similar estimates of *K*_d_ (Fig. 3). The 12% inter-assay coefficient of variation is at the upper end of what is commonly accepted in the literature, but we conclude that the saturation binding assay we set out here is sufficiently repeatable. It is critical to note, though, that the confidence intervals around a single *K*_d_ estimate generated by the non-linear regression do not indicate how close the estimate is to the ‘real’ *K*_d_. Confidence intervals from a single run are an indication of how precisely the *K*_d_ parameter can be estimated from the data; narrow confidence intervals mean that the data have a good ‘shape’, not that the real *K*_d_ of CBG has been determined precisely.

### Variation among individuals

Species vary in the amino acid sequence of CBG, and this variability is likely to be one cause of inter-specific variation in binding affinities (Hammond *et al*., 1991). Variability in *K*_d_, apparently caused by amino acid substitutions, has also been observed among breeds of pigs (Guyonnet-Dupérat *et al*., 2006) and among CBG-variant humans (Gagliardi *et al*., 2010; Lin *et al*., 2010). Glycosylation of CBG may also affect binding properties of CBG (Avvakumov and Hammond, 1994; Lin *et al*., 2010) and, in rats, the number and types of sugars attached to individual CBG molecules have been found to be highly variable (Ali and Bassett, 1995). It is therefore possible for CBG binding affinity to vary among individuals and modestly within individuals during such states as pregnancy (Hammond, 1990). Most researchers rely on pooled plasma samples when determining *K*_d_, but because there is the potential for inter-individual variation, we decided to measure *K*_d_ using individual plasma samples. The variation in measured *K*_d_ values among individuals can be attributed to both inter-assay variation and to real biological differences in *K*_d_. There was more variation among the *K*_d_ values calculated from individual Richardson's ground squirrels (ranging from 2.0 to 7.3 nM; coefficient of variation of 33%) than there was in inter-assay variation from our red squirrel plasma pools (coefficient of variation 12%), suggesting the possibility that there is real variation in CBG binding characteristics among individuals. There are other explanations for why these individual *K*_d_ values were more variable than the inter-assay variation measured in our red squirrel experiment. First, the Richardson's ground squirrel plasma samples were inherently more variable in the amount of CBG, albumin and other plasma proteins than the uniform red squirrel plasma pool. It is possible that these slight differences in assay conditions led to higher inter-assay variation. The Richardson's ground squirrel plasma had also been through different numbers of freeze–thaw cycles, which could affect binding characteristics.

Nevertheless, even if the observed differences in binding affinity were genuine biological differences, the question remains whether the observed differences have any biological significance. We addressed this question by comparing how the almost 4-fold difference in *K*_d_ values affected free hormone levels using data on total cortisol and maximal GC binding capacities from our previous studies. We calculated how much free cortisol levels would differ based on the maximal difference in *K*_d_ values. Figure 5 shows the relative effects of increasing *K*_d_ from 2.0 to 7.3 nM (detailed in Supplementary Appendix 2).

The animals with the highest percentage change in free cortisol (>200%) were almost exclusively animals with low total cortisol levels and high binding capacity, resulting in free cortisol levels <2 ng ml^−1^ when *K*_d_ = 7.3 nM. In theory, it is possible for very low GC concentrations to be meaningful biologically, especially in baseline conditions (i.e. in the absence of acute stressors). However, because Richardson's ground squirrels often have free cortisol levels >50 ng ml^−1^ (Fig. 5), even in baseline conditions (Delehanty and Boonstra, 2012), we conclude that there is likely to be no biological significance to the difference in free cortisol concentrations when animals have low cortisol and high binding capacity; that is, even if our observed range of *K*_d_ values represents real variation in CBG among individuals, the variation is likely to have no biologically relevant effect for these high-binding-capacity, low-cortisol animals.

The animals for which the increase in *K*_d_ increased free cortisol levels >10 but <164% were the animals that showed the greatest absolute change in free cortisol levels (4–8 ng ml^−1^). These tended to be animals that had binding capacities approximately equal to their total cortisol levels (Supplementary Table S2). Although these are not large differences compared with the range of free cortisol levels found in Richardson's ground squirrels over a wide range of stress levels, it is not easy to dismiss them as inconsequential without further investigation, especially as 19 of 96 animals fell into this category. In these animals, using a mean *K*_d_ based on a plasma pool would hide the fact that two individuals with the same total cortisol levels and binding capacities may genuinely have meaningfully different free cortisol levels.

For the rest of the animals (Supplementary Table S2), increasing *K*_d_ from 2.0 to 7.3 nM increased free cortisol levels <10%. All of these animals had high free cortisol concentrations at *K*_d_ = 2.0 nM (>50 ng ml^−1^), and the changes in free cortisol in absolute and percentage terms were both very low. This is because high free cortisol levels in these animals were the result of total cortisol levels exceeding binding capacity by a considerable amount (>30%). In these conditions, the existing high proportion of free cortisol simply overwhelms the effect of a change in binding affinity. In these animals, we conclude that individual variation in *K*_d_ is unlikely to have any biological relevance.

We conclude that it is possible that Richardson's ground squirrels show variation in *K*_d_ values that may be biologically meaningful in at least a subset of Richardson's ground squirrels. Although the evidence is preliminary, it at least warrants future study. In field studies of wild animals, though, once the experimental errors in measuring cortisol levels, binding capacities and *K*_d_ are considered, trying to use individual *K*_d_ values to calculate free hormone levels is unlikely to be precise enough to justify the considerable effort that would be required. It may be worth measuring the impact of individual variation in *K*_d_ values in more controlled laboratory studies of wild animals. In particular, because differences in *K*_d_ have the greatest effect at low free GC levels, studies looking at how baseline GC levels affect precisely measurable variables, such as foraging behaviour or parental care, might gain the most from incorporating individual *K*_d_ measurements.

### Variation among mammalian species

**Figure 6:**
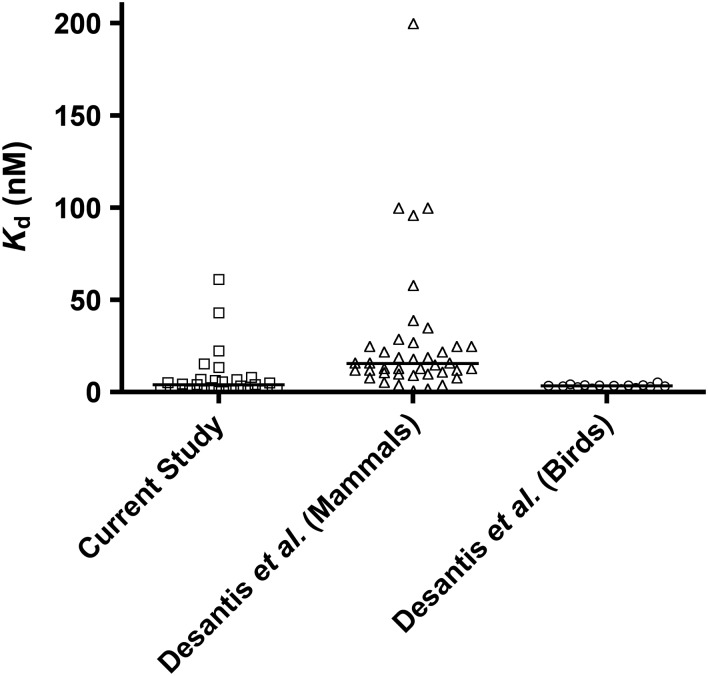
Distribution of *K*_d_ values from the present study and from those catalogued by Desantis *et al*. (2013). The horizontal bar represents the median value. In the present study, where we had multiple determinations for a single species we present the mean value. For the studies catalogued by Desantis *et al*. (2013), where there were multiple determinations for a single species from different studies, we have plotted each determination separately instead of averaging because the saturation binding techniques varied and estimates were sometimes divergent. For mammalian studies, there were 42 determinations in 35 species. For birds, there were 16 determinations of 13 species.

The majority (23 of 28) of species in our eclectic sample had *K*_d_ values clustered between 0.4 and 8.0 nM (median = 4.0 nM), with the five remaining species having *K*_d_ values ranging from 13 to 61 nM (Fig. 6). In a recent review that compared free hormone levels in a wide cross-section of species, Desantis *et al*. (2013) reported 61 *K*_d_ values for 51 species (some had multiple determinations). In mammals, the distribution of *K*_d_ values in the 42 determinations of 35 species was similar to ours. Most species had relatively low values (from 0.8 to 40 nM, with a median value of 16 nM), but there was a similar tail of species with higher values (up to 200 nM, with some species having questionable values of up to 500 nM). Thus, in these latter species, binding of GC was much weaker than the average. Notably, Desantis *et al*. (2013) also reported on the apparent absence of CBG binding capacity in two species of flying squirrels (*Glaucomys* spp.), which is also apparent in some New World primates. Fewer data were available for birds, but the *K*_d_ values appear to be more constrained, with the 16 *K*_d_ determinations in 13 species of birds all falling within a narrow range of 1.5–5.1 nM (i.e. they all bind GC strongly and consistently relative to mammals). There are a number of published *K*_d_ determinations for birds that have yielded unusually low (<8 pM; Wingfield *et al*., 1992) or high *K*_d_ values (>450 nM; Romero *et al*., 1998a, b, c) that are unlikely, we think, to be accurate, owing to the use of Scatchard plots. We have not included them here.

These data raise two interesting questions for further investigation. First, mammals appear to have more variable binding affinities than do birds (including some mammalian species that have essentially no binding capacity; Klosterman *et al*., 1986; Desantis *et al*., 2013). Given that there are fewer *K*_d_ values reported for avian species, it may be pure chance that the known species fall within a constrained range. Alternatively, the greater range of mammalian *K*_d_ values could reflect greater variability in techniques used by mammalian researchers (virtually all bird studies reported by Desantis *et al*., 2013 used vacuum fitration over glass fibres). However, if this pattern is real, then the second question that arises is whether there is a biological function of weaker binding affinities (i.e. high *K*_d_ values) that pertains to some mammals but not birds, or do mammals with weak binding affinity simply compensate by having more CBG?

Another important feature of our data is that the *K*_d_ values calculated here differ considerably from those using microdialysis methods. We have previously calculated the *K*_d_ for Arctic ground squirrels, snowshoe hares and meadow voles (Boonstra and Tinnikov, 1998; Boonstra *et al*., 2001, 2005). In all cases, the *K*_d_ for the microdialysis method was higher than the *K*_d_ using the charcoal method as described here (Arctic ground squirrel, 22 nM by microdialysis and 4 nM by charcoal; snowshoe hare, 20 nM by microdialysis and 0.6 nM by charcoal; and meadow voles, 16 nM by microdialysis compared with our value of 5 nM). This is somewhat surprising because the microdialysis assays were performed at 4°C, whereas in the charcoal method we equilibrated samples at 37°C. Affinity should be lower (*K*_d_ higher) at higher temperatures (e.g. Klosterman *et al*., 1986). One explanation may be that the microdialysis method did not take into account the presence of albumin, and that may have affected the calculation of *K*_d_. Confirming this possibility would require specific investigation. Another potential factor is that there may be geographical variation in *K*_d_ values (Breuner *et al*., 2003). The Arctic ground squirrel and meadow vole plasma used in the two types of assays were collected from distinct populations, separated by at least several hundred kilometres, so geographical variation in *K*_d_ could be a factor in addition to or instead of assay method. The possibility of geographical variation is further supported by the fact that our mean *K*_d_ of 61 nM for red squirrels was calculated based on animals collected from the Yukon, whereas the *K*_d_ of 29 nM was for animals from southern Ontario (Desantis *et al*., 2013). A rigorous comparison of separation methods for saturation binding assays using a common plasma pool would be a valuable contribution to the comparative literature.

### Conclusions

Measurement of free hormone levels in the study of stress in wildlife is critical (Breuner *et al*., 2013), and to do that one must know the *K*_d_ of the study species and then measure the maximal GC-binding capacity of individual blood samples. Saturation binding analysis with charcoal separation provides a fast and easily achievable method for *K*_d_ calculation that does not require expensive equipment, and it allows one to estimate the best parameters for making point estimates of maximal binding capacity (Hammond and Lähteenmäki, 1983). Although the assay must be optimized for each species, we were able to obtain good results over a range of plasma dilutions and hormone concentrations, and despite some ligand depletion. Nonetheless, we did encounter some situations in which we could not generate reliable saturation binding curves for reasons that we were not able to resolve. This is not necessarily a problem related to this technique, because others have encountered similar difficulties using other techniques (Klosterman *et al*., 1986).

Comparative physiologists need to incorporate CBG into their research routinely. Not only will this allow for the calculation of free hormone levels, but also it may shed light on the significance of variability in CBG binding affinity and capacity. Few broad trends have emerged from studies of how GCs are related to life-history traits, despite the immensely important role of GCs in allowing species to respond to their environments (Crespi *et al*., 2013). Corticosteroid-binding globulin is an important mediator of the biological effects of circulating GCs (Siiteri *et al*., 1982; Hammond, 1990; Perogamvros *et al*., 2011; Qian *et al*., 2011), so it may be that more patterns will emerge once we regularly incorporate the binding characteristics of CBG (including the dynamics of binding capacity and variability in affinity) into our studies of stress in wildlife.

## Supplementary material

Supplementary material is available at *Conservation Physiology* online.

## Funding

Funding for this research was from an NSERC Discovery Grant to R.B.

## Supplementary Material

Supplementary Data

Supplementary Data
